# Investigation of real-world heparin resistance and anticoagulation management prior to cardiopulmonary bypass: report from a nationwide survey by the Japanese Association for Thoracic Surgery heparin resistance working group

**DOI:** 10.1007/s11748-023-01936-5

**Published:** 2023-05-17

**Authors:** Koki Ito, Konosuke Sasaki, Minoru Ono, Takaaki Suzuki, Kisaburo Sakamoto, Hirotsugu Okamoto, Nobuyuki Katori, Naoki Momose, Yasuyuki Araki, Keiichi Tojo, Masahiro Ieko, Yutaka Komiyama, Yoshikatsu Saiki

**Affiliations:** 1 Division of Cardiovascular Surgery, Tohoku University Graduate School of Medicine, 1-1, Seiryomachi, Aoba-ku, Sendai, 980-8574 Japan; 2 Department of Cardiac Surgery, The University of Tokyo, Tokyo, Japan; 3 Department of Pediatric Cardiac Surgery, Saitama Medical University International Medical Center, Saitama, Japan; 4 Department of Cardiovascular Surgery, Mt. Fuji Shizuoka Children’s Hospital, Shizuoka, Japan; 5 Department of Anesthesiology, Kitasato University School of Medicine, Sagamihara, Japan; 6 Department of Anesthesiology, The Jikei University School of Medicine, Tokyo, Japan; 7 Department of Medical Center, Jichi Medical University, Saitama, Japan; 8 Department of Clinical Engineering, Saiseikai Kumamoto Hospital, Kumamoto, Japan; 9 Department of Medical Engineering, Kitasato University Hospital, Sagamihara, Japan; 10 Department of Hematology, Iwate Prefectural Chubu Hospital, Kitakami, Japan; 11 Faculty of Health and Medical Sciences, Hokuriku University, Kanazawa, Japan

**Keywords:** Heparin resistance, Activated clotting time, Cardiopulmonary bypass, Antithrombin concentrate, Cardiovascular surgery

## Abstract

**Objective:**

Heparin resistance is often encountered during cardiopulmonary bypass. Heparin dose and activated clotting time target values for the initiation of cardiopulmonary bypass are not yet universally standardized; further no consensus exists on the management of heparin resistance. This study aimed to investigate the current real-world practice on heparin management and anticoagulant treatment for heparin resistance in Japan.

**Methods:**

A questionnaire survey was conducted at medical institutions nationwide with which The Japanese Society of Extra-Corporeal Technology in Medicine members are affiliated, targeting surgical cases with cardiopulmonary bypass performed from January 2019 through December 2019.

**Results:**

Among 69% (230/332) of the participating institutions, the criterion for heparin resistance was defined as “the target activated clotting time value not reached even with an additional dose of heparin administration”. Cases of heparin resistance were reported in 89.8% (202/225) of the responded institutions. Of note, 75% (106/141) of the responded institutions reported heparin resistance associated with antithrombin activity ≥ 80%. Antithrombin concentrate was used in 38.4% (238/619 responses) or third dose of heparin in 37.8% (234/619 responses) for advanced heparin resistance treatment. Antithrombin concentrate was found to be effective in resolving heparin resistance in patients having normal, as well as lower antithrombin activity.

**Conclusion:**

Heparin resistance has occurred in many cardiovascular centers, even among patients with normal antithrombin activities. Interestingly, the administration of antithrombin concentrate resolved heparin resistance, regardless of the baseline antithrombin activity value.

**Supplementary Information:**

The online version contains supplementary material available at 10.1007/s11748-023-01936-5.

## Introduction

Heparin resistance (HR) is often encountered before the initiation of cardiopulmonary bypass (CPB), a prerequisite procedure for the majority of cardiovascular surgeries. HR is defined as the failure to achieve the desired activated clotting time (ACT), despite administration of clinically relevant dose of heparin. HR leads to extension of surgical time, because, CPB cannot be commenced promptly; further it may compromise appropriate anticoagulation throughout CPB, or result in a practical overdosing of heparin. However, there is no established consensus on the diagnosis of HR, and the exact mechanism behind HR is not yet clear. The reported frequency of HR range varies between 4 and 26% among the patients who underwent surgical procedures with CPB [1–7]. This variation in incidences might be attributable to the variations in the initial heparin doses administered and target ACT values employed at each institution. More importantly, no integrated therapeutic strategy for the anticoagulant management of HR has been established. Nevertheless, clinical practice management of HR varies; for example, the administration of antithrombin (AT) concentrate, fresh frozen plasma, or additional doses of heparin, and switching from heparin to argatroban, are employed [1]. Lobato et al. [8] reported the results of their survey conducted across 54 North American institutions in 2010, and a wide range of target ACTs (between 400 and 480 s) was documented. Similarly, based on the survey conducted among the members of the “Society of Cardiovascular Anesthesiologists” in 2019, Sniecinski et al. [9] reported that 74.9% of the respondents used empiric weight-based dosing of heparin, among which 70.7% of them targeted an ACT of either 400 or 480 s. In Japan, the “Report on Increased Pressure of Extra-Corporeal Membrane during Cardiovascular Surgery Using Cardiopulmonary Bypass” (from The Japanese Society for Cardiovascular Surgery Working Group for Increased Pressure of Extra-Corporeal Membrane) that was published in August 2016 described the recommended anticoagulant management and target ACT during cardiovascular surgery using CPB; however, till date, there are no reports that elucidate the real-world clinical practice. Thus, establishing a consensus on the definition of HR and integrated anticoagulant management to properly treat HR will be extremely useful. The institutional review board approved this study protocol (2022-1-556).

### Objective

This study aimed to elucidate the current clinical practices regarding heparin dosing and anticoagulant management for HR in Japan.

## Methods

### Survey distribution

We conducted a survey between January and December 2019 to acquire the HR-associated data prior to cardiovascular operations with CPB at 537 medical institutions nationwide, affiliated with JaSECT members. The survey period lasted 3 months, from November 2020 to January 2021. The questionnaire consisted of ten primary questions, available on the JaSECT homepage to facilitate easy access to the questions.

### Handling of personal and facility information

Personal information that can potentially identify a specific individual, such as names and e-mail addresses, was only used for contact purposes of the survey. The questionnaire responses have been consolidated and subsequently analyzed. It was ensured that the responses have not been used for any other purposes. The recorded personal information was encrypted using Secure Sockets Layer (SSL) to ensure the confidentiality and security. Further, the facility information was deleted from the response data during the consolidation, and it was stored in a format that could not be identified from this data.

### Statistical analyses

Based on the number of target facilities, 537 institutions, to obtain the confidence intervals (CIs) of 95% and sample proportion of 0.5, we calculated that 284 responses would be needed to achieve a global margin of error of 4%. Descriptive statistics were used to summarize the data. Wald CIs for the proportions were set at 95%. All calculations were performed using JMP Pro 16 (SAS Institute Inc., Cary, NC, USA).

## Results

Responses from 341 (63.5%) of the 537 institutions were obtained and included in the analysis. If reply fields were left blank, the questions were counted as “no answer”. The number of CPB procedures performed at the responding institutions is summarized in Table 1.

**Table 1:** A summary of the survey responses

Demographics of respondents	n (%)	
Number of CPB cases per year		
≤50	98 (28.8)	
51–100	105 (30.9)	
101–200	92 (27.1)	
201–300	33 (9.7)	
> 300	12 (3.5)	
Number of CPB cases per year in adults		
≤50	105 (32.0)	
51–100	105 (32.0)	
101–200	85 (25.9)	
201–300	26 (7.9)	
> 300	7 (2.1)	
ACT target value for initiation of CPB (seconds)	n (%)	95% CI
< 400	5 (1.5)	0.6–3.5
400	99 (29.6)	25.0–34.7
401–479	21 (6.3)	4.1–9.4
480	175 (52.4)	47.0–57.7
> 481	34 (10.2)	7.4–13.9
Heparin management		
Initial dose of heparin (IU/kg)	n (%)	95% CI
< 300	44 (13.3)	10.1–17.4
300–349	237 (71.8)	66.7–76.4
350–399	8 (2.4)	1.2–4.7
≥400	41 (12.4)	9.3–16.4
Attitudes of heparin resistance		
Definition of heparin resistance	n (%)	95% CI
Target ACT value was not reached with initial heparin administration	65 (19.6)	15.7–24.2
Target ACT value was not reached with an additional dose of heparin administration	230 (69.3)	64.1–74.0
Others	37 (11.1)	8.2–15.0
Number of additional heparin administrations allowed before CPB		
1 time	104 (47.1)	40.8–53.9
2 times	97 (43.9)	37.2–50.2
3 times	17 (7.7)	4.9–12.0
4 times	1 (0.5)	0.1–2.5
≥ 5 times	2 (0.9)	0.2–3.3
Have you experienced HR patients according to each criterion?	n (%)	
No	23 (10.2)	
Yes	202 (89.8)	
Frequency of HR occurrences in adult CPB patients	n (%)	95% CI
0%	23 (10.2)	6.9–14.9
< 10%	133 (59.1)	52.6–65.3
≥10%, < 20%	39 (17.3)	12.9–22.8
≥20%	30 (13.3)	9.5–18.4
Further treatment for HR		
AT concentrate	238 (38.4)	34.7–42.3
Additional heparin	234 (37.8)	34.1–41.7
Argatroban	60 (9.7)	7.6–12.3
Nafamostat mesylate	43 (6.9)	5.2–9.2
Fresh frozen plasma	40 (6.5)	4.8–8.7
Others	4 (0.6)	0.3–1.6

Data are given as n (%) of respondents selecting a particular answer

*CPB* cardiopulmonary bypass, *ACT* activated clotting time, *HR* heparin resistance, *AT* antithrombin

### ACT target value and heparin management

The details of ACT measuring devices were shown in Supplemental Table, 29% (105/364) of the institutions used the Hemochron^®^ Signature Elite (International Technidyne Corporation, Edison, NJ, USA), and 27% (100/364) of the institutions used Hemochron^®^ Response (International Technidyne Corporation, Edison, NJ, USA); Of the institutions that responded, 52% (175/334) kept the target ACT value as 480 s. The usual initial dose of heparin was set at “300–349 IU/kg” during the CPB surgery in 72% (237/330) of the institutions. If the target ACT was not reached after the initial administration of heparin, an additional dose was administered. The total required dose of heparin (including the initial dose), that was judged to be compatible in the event of HR, was estimated to be approximately 400 IU/kg in 32% (66/208) of the institutions (Table 1).

### Definition of heparin resistance

The criterion for determining HR varies across institutions, and 69% of the institutions defined HR as a condition where the “target ACT value was not reached despite administration of additional dose of heparin”. One additional heparin dose was permissible in 47% (104/221) of the institutions, and two additional doses in 44% (97/221) of the institutions. The patients in 90% (202/225) of the institutions showed HR according to each criterion. Additionally, the frequencies of HR occurrences in adult CPB patients were “less than 20% (excluding 0%)” at 76% (172/225) of the institutions (Table 1).

### Management of heparin resistance using anticoagulants

**Fig. 1:**
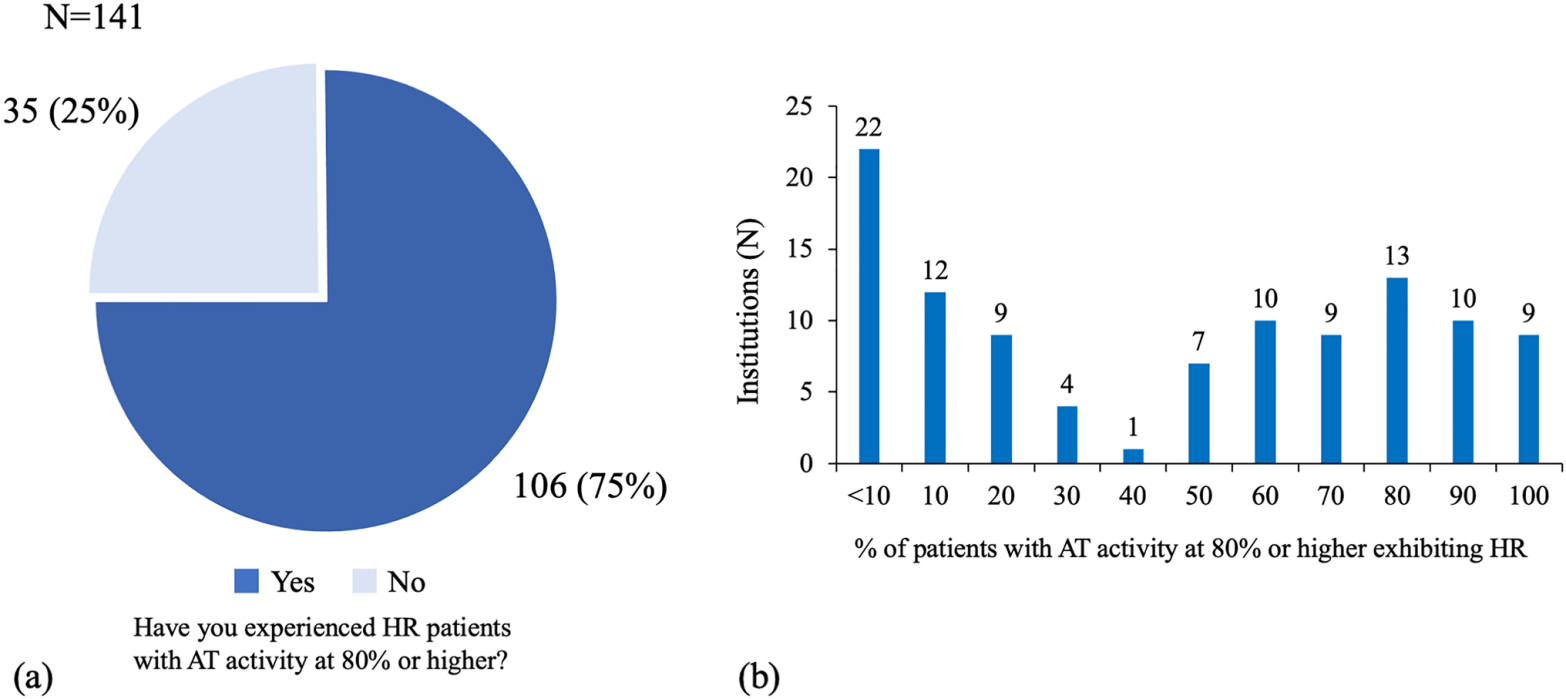
The proportion of patients with AT activities at 80% or higher in HR cases. **a** The pie graph shows 106 institutions (75%) answered yes and 35 (25%) answered no to the question “Have you ever experienced HR patients with AT activity at 80% or higher?”. **b** The bar graph shows the detail of the number of institutions among those that answered “Yes” to the question (**a**). Each institution has experienced varying proportion of HR patients despite AT activity at 80% or higher. *AT* antithrombin, *HR* heparin resistance

**Fig. 2:**
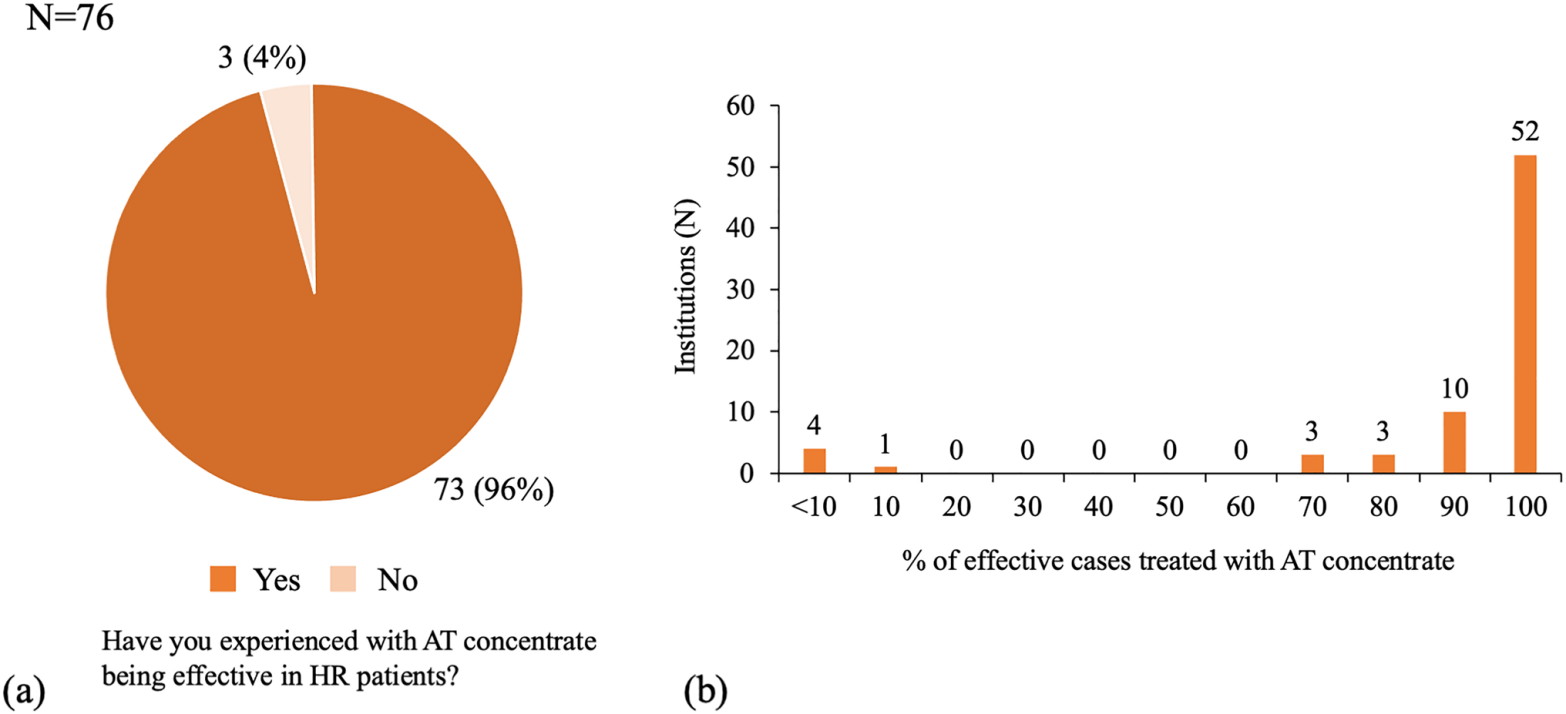
Observed efficacy of the AT concentrate in HR patients irrespective of the AT activity. **a** The pie graph shows 73 institutions (96%) answered yes and 3 (4%) answered no to the question “Have you ever experienced with AT concentrate being effective in HR patients”. **b** The bar graph shows the detail of the number of institutions among those that answered “Yes” to the question (**a**). It depicts that most of the institutions have experienced 70% or higher effectiveness of AT concentrate in the cases treated for HR regardless of the baseline AT activity. *AT* antithrombin, *HR* heparin resistance

For the question on additional treatment for HR, the denominator (N = 619) was the sum of responses to first-line treatment (N = 326) and second-line treatment (N = 301), excluding 8 cases of whom surgery was cancelled. The additional treatments for HR included administration of AT concentrate (38%; 238/619 responses), additional administration of heparin (38%; 234/619 responses), and argatroban (10%; 60/619 responses). In 75% (106/141) of the institutions, HR patients were observed with baseline AT levels at 80%, or even higher (Fig. 1a). Among institutions experiencing HR patients with normal AT activity levels, the proportion of those cases to total HR patients varied (Fig. 1b). Among the institutions that administered the AT concentrate to HR patients regardless of AT activity, 96% (73/76) of the institutions reported that AT concentrate was effective in HR patients (Fig. 2a), and in 93% (68/73) of those institutions, AT concentrate were effective in 70% or more of the total cases, where the participants achieved the target ACT (Fig. 2b). Administration of the AT concentrate to HR patients with recorded AT activities less than 80% (low AT activity value) was effective, as confirmed by 80% (24/30) of the institutions experiencing over 70% effective cases of the total. Among institutions where the AT concentrate was administered to the patients who exhibited HR even with the baseline AT activity value of 80% or higher, 82% (28/34) reported that 70% cases attained target ACT. For the acquisition of medical insurance reimbursements for AT concentrate administration (to treat HR) during cardiovascular surgery with CPB, 82% (261/317) of the institutions answered that they “need to acquire extended indication” (Supplemental Figure 1).

## Discussion

This report was created based on the analyses of the survey conducted by the task force members from four academic societies (The Japanese Association for Thoracic Surgery, The Japanese Society for Cardiovascular Surgery, The Japanese Society of Cardiovascular Anesthesiologists, and The Japanese Society of Extra-Corporeal Technology in Medicine). The total number of cases of CPB use in 2019 at the institutions that responded to this survey was 37397, accounting for 69% (37397/54047) of the total in Japan. Our study revealed that there are significant variations in the current clinical practices in Japan with regard to heparin dosing, target ACT values, diagnosis of HR, and anticoagulation treatment for HR in cardiovascular surgeries with CPB. We found that an initial heparin dose of 300 IU/kg was predominantly used by practitioners to obtain the target ACT value of 480 s. This dosing is consistent with the recommendations in “Report on Increased Pressure of Extra—Corporeal Membrane During Cardiovascular Surgery Using Cardiopulmonary Bypass” (The Japanese Society for Cardiovascular Surgery Working Group for Increased Pressure of Extra-Corporeal Membrane). When the criterion for HR was defined, as stated in this study, many institutions reported HR cases. The anticoagulant treatments have varied across institutions till date. In fact, many institutions utilized AT concentrate even without reimbursements by health insurance for that specific indication, as previously reported [10–14].

The mechanisms underlying HR have not been fully understood, because, they are complex, multi-factorial, and intricately related. However, interestingly, this survey unleashed the various factors involved in HR, such as low AT activity, preoperative exposure to heparin, heterogeneity in the titer of heparin preparation, inflammation and infections, and the presence of heparin-binding plasma protein. AT deficiency has long been hypothesized as the primary mechanism of HR, because, heparin’s anticoagulant effect is mediated indirectly via the AT pathway [2, 14, 15]. At deficiency is associated with variety of clinical conditions, including nephropathy [16, 17], liver disease [18–20], preoperative heparin treatment [4, 5, 21, 22], malnutrition, and mechanical circulatory support [4, 23, 24]. The results of this survey also indicated that the administration of AT concentrate (for low AT activity) effectively improved HR.

In contrast, several institutions reported cases exhibiting characteristic HR symptoms even when the baseline AT activities were within the standard value range. The main underlying mechanism associated with these HR cases may not be mediated by AT. An increase in the heparin-binding proteins and platelets has been speculated [1]. Heparin-binding proteins include chemokines such as platelet factor 4, extracellular matrix proteins, growth factors, enzymes such as superoxide dismutase and elastase, and miscellaneous such as factor VIII / von Willebrand factor and lipoproteins [1]. Additionally, the nonspecific binding of platelets to heparin and thrombophilia are also reported as factors linked to HR. There are multiple other potential mechanisms that can lead to HR, which are both poorly defined and nonspecific. It must also be considered that the ACT itself may not accurately reflect the anticoagulant activity of heparin. ACT is indeed affected by multiple variables, many of which are commonly seen during cardiac surgery with CPB such as hypothermia, blood dilution, preoperative warfarin and antiplatelet use, and platelet count. The patient’s high age (> 65 years old), preoperative administration of heparin, increased platelet count, increased levels of heparin-binding protein and factor VIII, and endocarditis are risk factors attributed to HR emergence [25]. In addition, Kawatsu et al. [26] reported that high preoperative fibrinogen levels, smoking habit, preoperative diagnosis of chronic aortic dissection, and chronic obstructive pulmonary disease were associated with increased possibility of HR occurrence.

Our most interesting finding was that 82% of the institutions perceived the efficacy of AT concentrate in 70% or more of the patients administered with the AT concentrate, despite their baseline AT activities exceeding 80% or more. One can speculate that the AT activity, as one of the clinical indices, is not necessarily reflective of the capacity for anticoagulants, despite the absence of reports about the mechanisms of this phenomenon. Nevertheless, these results strongly suggest the significant clinical efficacy of AT concentrate for HR cases. Further investigations are warranted to elucidate the underlying mechanism.

It should be mentioned that there were several limitations to this study. First, this study was based on the responses to a questionnaire survey and not on actual case or patient data. Similarly, this study was not an analysis of actual laboratory data itself, such as AT activity values. Secondly, the data in this study was not based on the actual number derived from pre-determined patient cohort, and there may be room for inaccuracy in some of the answers due to the inherent nature of this survey design. Thirdly, the response rate to the questionnaire survey was somewhat low (341/537: 63.5%), although it was much superior to those in the previous reports [8, 9]. Those factors may have affected the reliability of the response results. Therefore, 95% confidence intervals were appended to estimate the results of the responses as a population.

## Conclusion

The incidences of HR have been reported at majority of the cardiovascular centers, and even among the patients with normal AT activities. The administration of AT concentrate resolved HR regardless of the baseline AT activity.

### Supplementary Information

Below is the link to the electronic supplementary material.

Supplementary file1 (PDF 103 KB)

Supplementary file2 (PDF 159 KB)

Supplementary file3 (PDF 63 KB)

## Data Availability

The datasets generated and analyzed during the current study are available from the corresponding author on reasonable request.

## Abbreviations

ACT: Activated clotting time
AT: Antithrombin
CPB: Cardiopulmonary bypass
HR: Heparin resistance
JaSECT: Japanese Society of Extra-Corporeal Technology in Medicine
SSL: Secure sockets layer
