# Supplementary material for: Investigation of real-world heparin resistance and anticoagulation management prior to cardiopulmonary bypass: report from a nationwide survey by the Japanese Association for Thoracic Surgery heparin resistance working group
Source: Gen Thorac Cardiovasc Surg. 2023 May 17;72(1):8–14. doi: 10.1007/s11748-023-01936-5 (PMC10766675; doi:10.1007/s11748-023-01936-5)
Supplement: Supplementary file 1 — Supplementary file1 (PDF 103 KB) [file 11748_2023_1936_MOESM1_ESM.pdf]

Journal name: General Thoracic and Cardiovascular Surgery

Title: Investigation of Real-World Heparin Resistance and Anticoagulation Management Prior to Cardiopulmonary Bypass: Report from a Nationwide Survey by The Japanese Association for Thoracic Surgery Heparin Resistance Working Group

Koki Ito, MD, PhD[1]; Konosuke Sasaki, PhD[1]; Minoru Ono, MD, PhD[2]; Takaaki Suzuki, MD, PhD[3]; Kisaburo Sakamoto, MD, PhD[4]; Hirotsugu Okamoto, MD, PhD[5]; Nobuyuki Katori, MD, PhD[6]; Naoki Momose, CCP[7]; Yasuyuki Araki [8]; Keiichi Tojo[9]; Masahiro Ieko[10]; Yutaka Komiyama[11]; Yoshikatsu Saiki, MD, PhD[1]

[1]Division of Cardiovascular Surgery, Tohoku University Graduate School of Medicine, Sendai, Japan.

[2]Department of Cardiac Surgery, The University of Tokyo, Tokyo, Japan.

[3]Department of Pediatric Cardiac Surgery, Saitama Medical University International Medical Center, Saitama, Japan. [4]Department of Cardiovascular Surgery, Mt. Fuji Shizuoka Children's Hospital, Shizuoka, Japan.

[5]Department of Anesthesiology, Kitasato University School of Medicine, Kanagawa, Japan.

[6]Department of Anesthesiology, The Jikei University School of Medicine, Tokyo, Japan.

[7]Department of Medical Center, Jichi Medical University, Saitama, Japan.

[8]Department of Clinical Engineering, Saiseikai Kumamoto Hospital, Kumamoto, Japan.

[9]Department of Medical Engineering, Kitasato University Hospital, Kanagawa, Japan. [1]

[10]Department of Hematology, Iwate Prefectural Chubu Hospital, Kitakami, Japan.

[11]Faculty of Health and Medical Sciences, Hokuriku University, Kanazawa, Japan.

Address for Correspondence:

Yoshikatsu Saiki, MD, PhD

Division of Cardiovascular Surgery, Tohoku University Graduate School of Medicine

1-1, Seiryomachi, Aoba-ku, Sendai 980-8574, Japan.

Tel: (+81)-22-717-7222; Fax: (+81)-22-717-7227

E-mail: yoshisaiki@med.tohoku.ac.jp

Supplemental Figure Legends

Supplemental Figure 1. The demand acquisition of indications for medical insurance reimbursements for AT concentrates for HR patients

This pie graph illustrates that 82% (261 / 317) of the institutions answered that the acquisition of indications for medical insurance reimbursement for the use of AT concentrates for heparin resistance patients is necessary. AT, antithrombin; HR, heparin resistance.
