# Supplementary material for: Investigation of real-world heparin resistance and anticoagulation management prior to cardiopulmonary bypass: report from a nationwide survey by the Japanese Association for Thoracic Surgery heparin resistance working group
Source: Gen Thorac Cardiovasc Surg. 2023 May 17;72(1):8–14. doi: 10.1007/s11748-023-01936-5 (PMC10766675; doi:10.1007/s11748-023-01936-5)
Supplement: Supplementary file 2 — Supplementary file2 (PDF 159 KB) [file 11748_2023_1936_MOESM2_ESM.pdf]

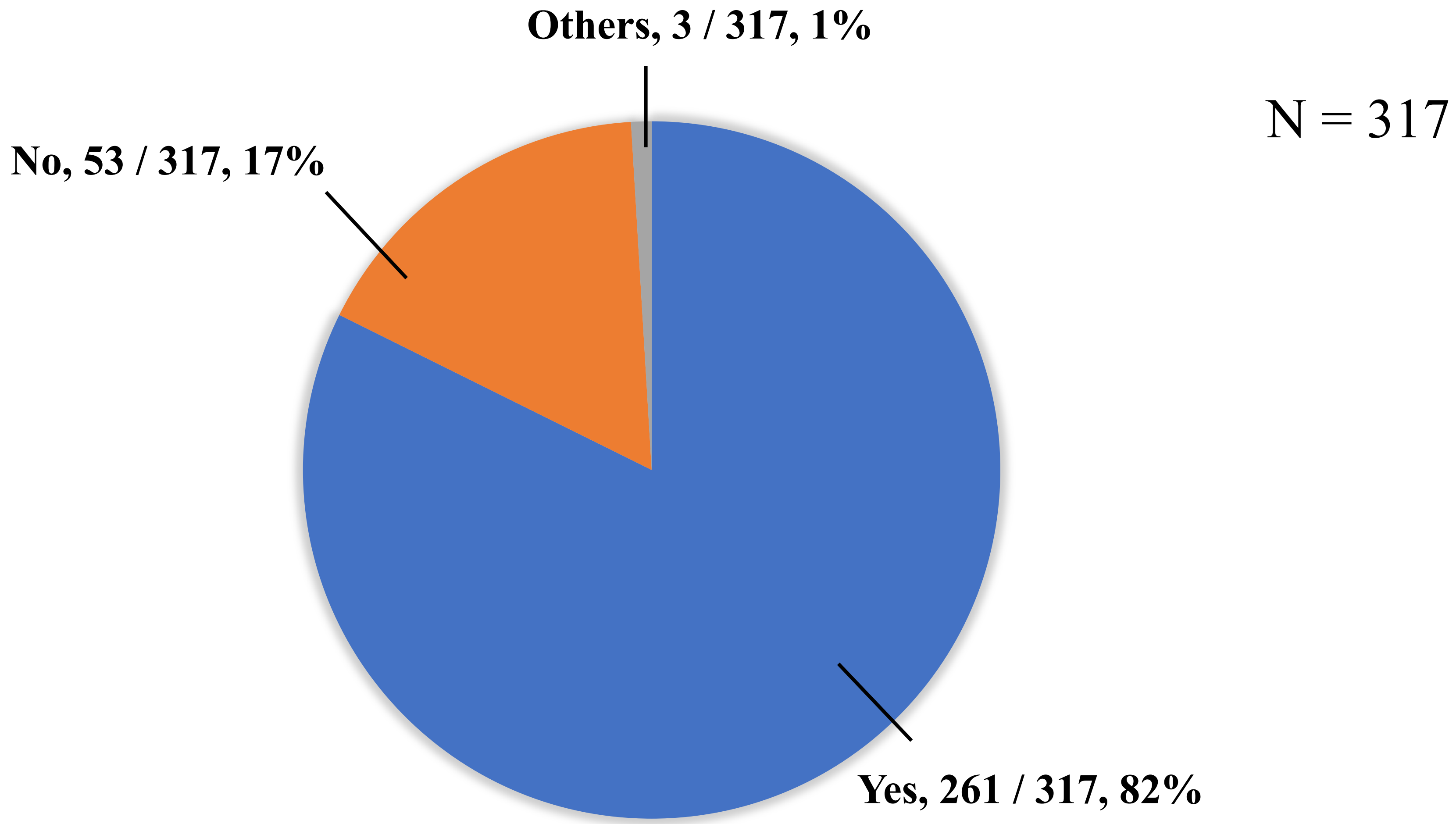

Is it necessary to obtain insurance indication of AT concentrate for heparin resistance?

Supplemental figure 1
