# Supplementary material for: Investigation of real-world heparin resistance and anticoagulation management prior to cardiopulmonary bypass: report from a nationwide survey by the Japanese Association for Thoracic Surgery heparin resistance working group
Source: Gen Thorac Cardiovasc Surg. 2023 May 17;72(1):8–14. doi: 10.1007/s11748-023-01936-5 (PMC10766675; doi:10.1007/s11748-023-01936-5)
Supplement: Supplementary file 3 — Supplementary file3 (PDF 63 KB) [file 11748_2023_1936_MOESM3_ESM.pdf]

Supplemental Table. A summary of the survey responses.

**ACT monitoring**	
ACT machine (including duplicate answer)	**n (%)**
Hemochron® Signature Elite	105 (28.8)
Hemochron® Response	100 (27.5)
Actalyke® MINI II	57 (15.7)
HMS Plus	29 (8.0)
Hemochron® 401	28 (7.7)
ACT Plus	21 (5.8)
Hemochron® Jr.	11 (3.0)
Others	13 (3.6)

Data are given as n (%) of respondents selecting a particular answer.
ACT, activated clotting time.
